# Supplementary material for: Postpartum breast cancer has a distinct molecular profile that predicts poor outcomes
Source: Nat Commun. 2021 Nov 3;12:6341. doi: 10.1038/s41467-021-26505-3 (PMC8566602; doi:10.1038/s41467-021-26505-3)
Supplement: Supplementary file 1 — Supplementary Information [file 41467_2021_26505_MOESM1_ESM.pdf]

Supplemental figure 1

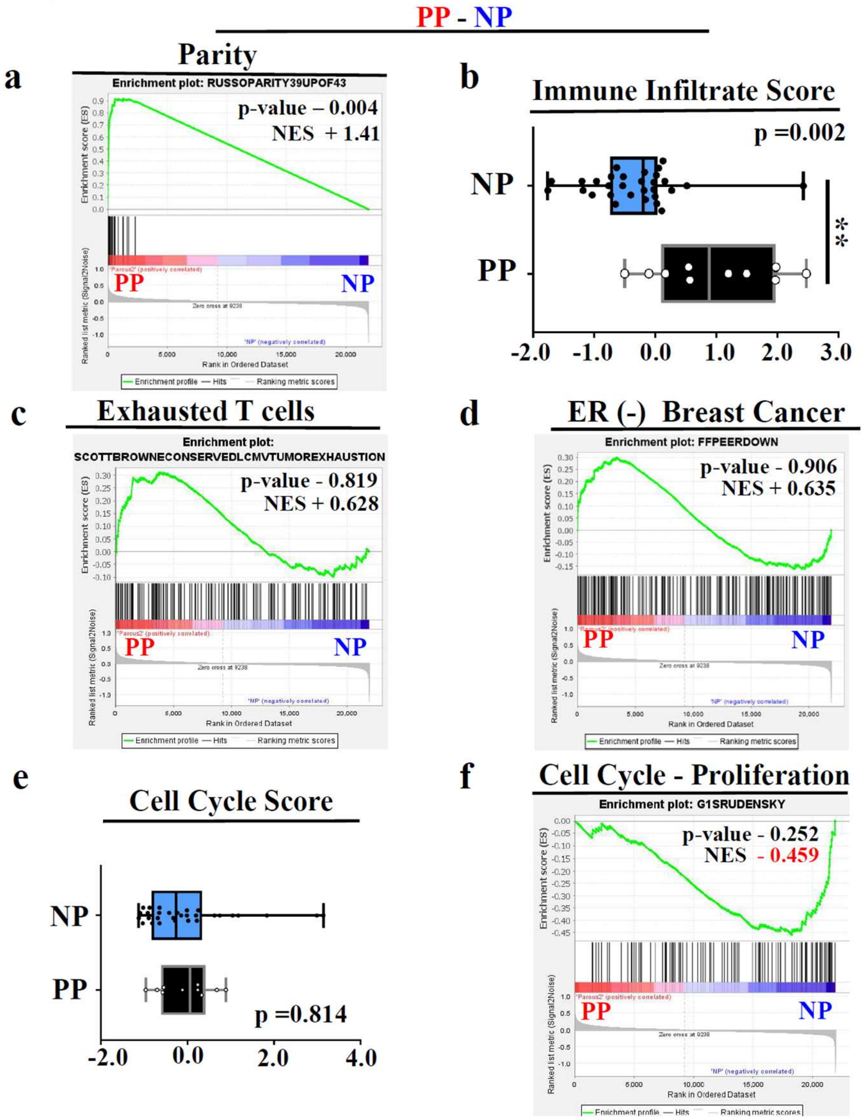

Supplementary Figure 1 RNA expression comparison between postpartum and nulliparous breast tissue in healthy normal women: Whole exome gene expression array data from healthy nulliparous (NP, blue, n = 30) or postpartum breast (PP, red, within 2 years of completed pregnancy, n= 10) tissues was obtained from a previous study (GEO-GSE26457). Data was normalized utilizing Transcriptome Analysis Console software (V.4.02, ThermoFisher Scientific). Gene set expression profiles or single sample scores for **a** parity (as if Fig 2f), **b** immune infiltrate (as in Fig 4a), **c** immune exhaustion (as if Fig 4g), **d** ER negative breast cancer **(**as in Fig 2d), **e** cell cycle (as in fig 3b) and **f** cell cycle (as in Fig 2a) are displayed. For enrichment plots p-values reported are nominal p-values reported by GSEA software with accompanying normalized enrichment scores (NES), while single sample p-values are from students' unpaired two-tailed t-test with Welch correction. For box and whisker plots (b,e), data are presented as minimum to maximum with median value marked by a line within the depicted interquartile range.

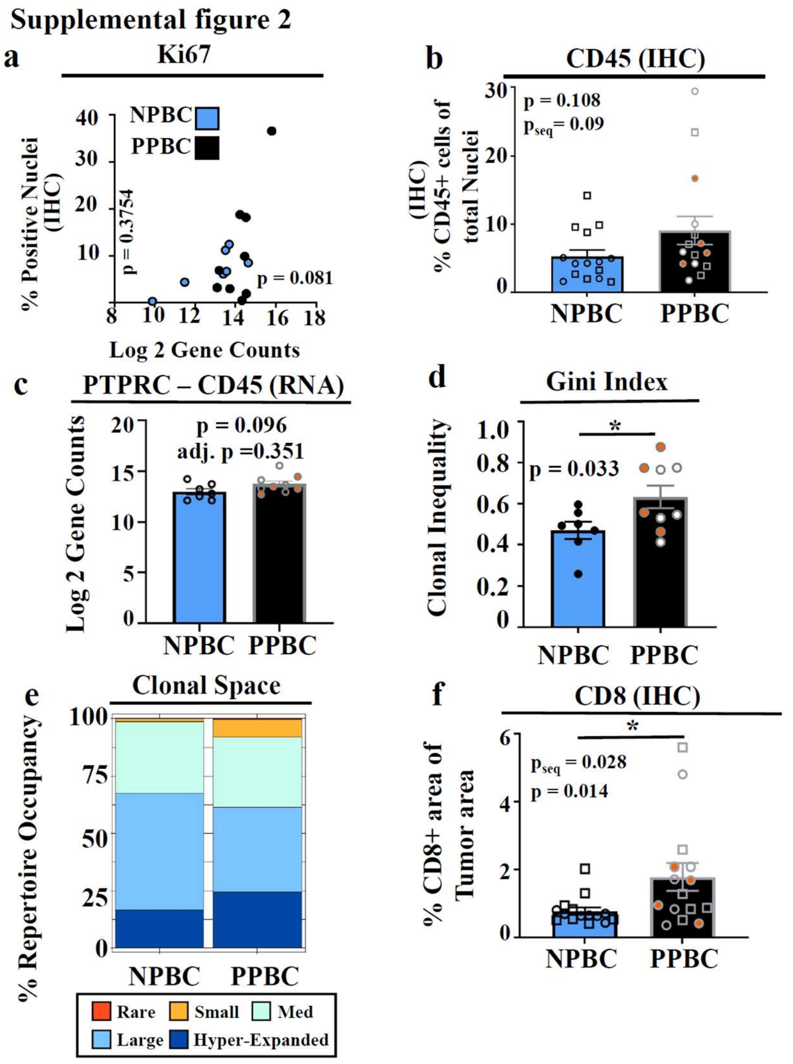

Supplementary Figure 2 Proliferation, inflammation, CD8 T cell and T cell clonality comparison between PPBC and NPBC: a Correlations between Ki67 gene expression levels by RNA seq and Ki67 protein expression by IHC illustrating overall concordance between protein and RNA, but no statistical significant difference between PPBC (n = 9) and NPBC (n = 7). P-values are displayed on corresponding axis and are computed based upon students' unpaired two-tailed t-test with Welch correction. **b** Semi quantitative immunohistochemical analysis of CD45 protein expression, (PPBC n = 15, NPBC n = 15) with p values determined by students' unpaired two-tailed t-test with Welch correction **c** RNA expression analysis of pan-immune expression protein/gene (CD45/PTPRC) between NPBC (n = 7) and PPBC (n = 9) shows no significant difference, p values determine by students' unpaired two-tailed t-test with Welch correction without and with adjustment for multiple comparisons (adj p) . **d** Economic inequality modeling based evaluation of normalized T cell clonality (Gini Index) demonstrates enhanced clonality within PPBC cases (n = 9) compared to NPBC (n = 7). **e** Relative make up of T-cell clonal expansion in PPBC (n = 9) or NPBC (n = 7) cohorts expressed as clonal space occupancy: rare (red), small (orange), med (light blue), large (blue) or hyper-expanded (dark blue) clones **f** CD8+ protein staining by IHC evaluated as percent positive signal of tumor area. P-values determined by students' unpaired two-tailed t-test with Welch correction (* p≤ 0.05), or students' unpaired one-tailed t-test with Welch correction for confirmatory IHC (PPBC n = 15, NPBC n = 15). Data depicted in bar graphs are presented as mean values +/- SEM. Samples

evaluated by RNA Seq are depicted by circles and Pseq refers to p-values derived from students' unpaired two-tailed t-test with Welch correction for these samples only (PPBC n = 9, NPBC n = 7). ICGC identified P53mutations are noted by orange filled circles.

Supplemental figure 3

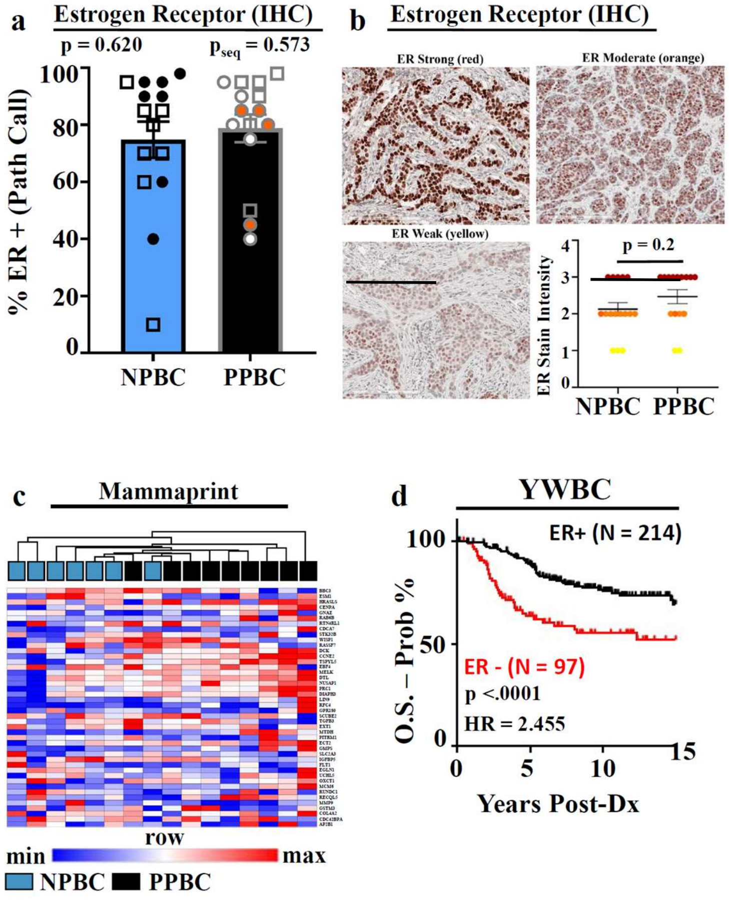

Supplementary Figure 3 Estrogen Receptor Expression Evaluation and Mammaprint gene clustering comparisons between PPBC and NPBC. Overall survival probability of compiled Young Women's Breast Cancer cohort by ER status: All 30 cases (n =15 NPBC, n =15 PPBC) were stained and evaluated for estrogen receptor expression by IHC. **a** Percent tumor ER positive nuclei (brown) was assessed by pathologist screening and recorded with no difference being observed between PPBC and NPBC cases in the RNA seq subset or complete data set. Data are presented as mean values +/- SEM. P-values were determined by students' unpaired two-tailed t-test with Welch's correction. Samples evaluated by RNA Seq are depicted by circles and Pseq refers to p-values derived from students' unpaired two-tailed t-test with Welch correction for these samples only (PPBC n = 9, NPBC n = 7). **b** examples of ER staining intensity and depiction of staining intensity distribution between cohorts (NPBC n = 15, PPBC n = 15) Data are presented as mean values +/- SEM. P-values were determined by students' unpaired two-tailed t-test with Welch's correction. Scale bar = 200um. **c** The gene expression values for the forty four genes of Mammaprint® found to be expressed above background levels in these samples were permitted to cluster based upon Z-score transformed Euclidean average linkage parameters, with no clear cluster based upon parity status observed (postpartum breast cancer, PPBC, black, n = 9) or nulliparous breast cancer(NPBC, blue, n = 7). **d** Gene expression profiles for 311 women under the age of 45 at time of a primary breast cancer diagnosis were obtain across seven studies to compile a Young Women's Breast Cancer (YWBC) cohort. Within this cohort survival probability was assessed when separated by clinically determine ER status (positive-black n = 214, negative-red n = FF97). P-values for survival determine by two-tailed log-rank (Mantel-Cox) evaluation and reported with Log-rank Hazard Ratios.

Supplemental Table 1a: Clinical characteristics of samples used in RNA sequencing or IHC

RNA sample designation	Age at Dx	RACE	Ethnicity	BMI	Stage at Dx	ER (%) by IHC	ER (on IHC)	PR (on IHC)	Her-2 (on IHC)	Parity	PAM50 designation by RNA seq
S01	40	white	NH	29.37284	2A	40	pos	pos	neg	Nulliparous	Her2
S02	33	white	NH	23.159462	2A	40	pos	neg	pos	Postpartum	Basal
S03	44	Filipino	NH	27.981009	3A	95	pos	pos	neg	Nulliparous	Lum B
S04	34	white	NH	24.40488	3A	80	pos	pos	neg	Postpartum	Lum A
S05	36	white	NH	22.683304	2A	90	pos	pos	neg	Nulliparous	Lum A
S06	43	Black	NH	21.464417	2A	98	pos	neg	equiv	Nulliparous	Lum A
S07	36	white	NH	25.921567	2A	75	pos	pos	neg	Postpartum	Lum B
S08	34	white	NH	33.844468	2B	85	pos	pos	equiv	Postpartum	Lum B
S09	43	white	NH	25.165958	2A	60	pos	pos	equiv	Nulliparous	Lum B
S10	32	white	NH	25.676607	3C	45	pos	pos	missing	Postpartum	Her2
S11	30	Black	NH	40.197555	3A	90	pos	pos	neg	Nulliparous	Lum A
S12	35	American Indian	NH	53.168585	2B	90	pos	pos	neg	Postpartum	Lum A
S13	33	white	NH	31.77487	2B	85	pos	pos	discord	Postpartum	Lum A
S14	38	white	NH	29.986427	2B	95	pos	pos	neg	Nulliparous	Lum B
S15	37	white	NH	21.560711	2B	80	pos	neg	equiv	Postpartum	Lum B
S16	34	white	NH	33.548668	3A	95	pos	pos	neg	Postpartum	Lum B
IHC17	40	white	NH	30.893555	3C	85	pos	pos	neg	Nulliparous	NA
IHC18	45	white	NH	33.629132	2B	70	pos	pos	equiv	Nulliparous	NA
IHC19	38	white	Unknown	NA	2B	70	pos	pos	missing	Nulliparous	NA
IHC20	30	Chinese	NH	19.202653	2A	85	pos	pos	neg	Nulliparous	NA
IHC21	45	white	NH	30.69573	3C	60	pos	pos	neg	Nulliparous	NA
IHC22	39	white	NH	34.776131	2A	95	pos	pos	discord	Nulliparous	NA
IHC23	40	white	NH	26.545306	2A	10	pos	neg	pos	Nulliparous	NA
IHC24	44	white	NH	34.199011	2B	80	pos	pos	neg	Nulliparous	NA
IHC25	32	white	NH	22.919678	3A	90	pos	pos	neg	Postpartum	NA
IHC26	32	white	NH	24.543213	2B	50	pos	pos	neg	Postpartum	NA
IHC27	37	white	NH	23.144212	2A	80	pos	pos	neg	Postpartum	NA
IHC28	44	Black	NH	29.570878	2B	98	pos	pos	neg	Postpartum	NA
IHC29	45	white	NH	23.098571	2A	95	pos	pos	equiv	Postpartum	NA
IHC30	36	white	NH	27.365917	3C	95	pos	neg	pos	Postpartum	NA

NH = Non-Hispanic, Pos = Positive, Neg = Negative, NA = Not Available, equiv = Equivalent, Discord = discordant

Supplemental Table 1b: Clinical characteristic comparison on the postpartum and nulliparous breast cancer cohorts

Clinical characteristics	Nulliparous cancer	Postpartum breast cancer	P-value
**Overall N**	15	15	
**Age at dx, mean (SD)**	39.6 (±4.8)	35.6 (±4.0)	0.02
**Pre-menopausal**	15	15	
**BMI**	29.0	28.2	0.38
**Race**			
White	11	14	
Black	2	1	
Other	2	1	
**Stage**			
1	0	0	>0.9*
2	11	10	
3	4	5	
4	0	0	
**ER status**			
Pos	15	15	
Neg	0	0	
**PR status**			
Pos	13	12	>0.9*
Neg	2	3	
**HER2 status**			
Pos	1	2	0.35
Neg	9	8	
Discordant/equivocal	4	4	
missing	1	1	

P-value * is based on Kolmogorov-Smirnov test
