# Supplementary material for: Postpartum breast cancer has a distinct molecular profile that predicts poor outcomes
Source: Nat Commun. 2021 Nov 3;12:6341. doi: 10.1038/s41467-021-26505-3 (PMC8566602; doi:10.1038/s41467-021-26505-3)
Supplement: Supplementary file 3 — Description of Additional Supplementary Files [file 41467_2021_26505_MOESM3_ESM.pdf]

Description of Additional Supplementary Files

File Name: Supplementary Data 1
Description: Annotated GSEA Gene Dataset GMX Matrix

File Name: Supplementary Data 2
Description: Full Results of Cibersort Analysis performed for Figures 4E and 4F
