# Supplementary material for: Postpartum breast cancer has a distinct molecular profile that predicts poor outcomes
Source: Nat Commun. 2021 Nov 3;12:6341. doi: 10.1038/s41467-021-26505-3 (PMC8566602; doi:10.1038/s41467-021-26505-3)
Supplement: Supplementary file 6 — Reporting Summary [file 41467_2021_26505_MOESM6_ESM.pdf]

nature research

Corresponding author(s): Pepper Schedin

Last updated by author(s): 09/27/2021

Reporting Summary

Nature Research wishes to improve the reproducibility of the work that we publish. This form provides structure for consistency and transparency in reporting. For further information on Nature Research policies, see our Editorial Policies and the Editorial Policy Checklist.

Statistics

For all statistical analyses, confirm that the following items are present in the figure legend, table legend, main text, or Methods section.

n/a	Confirmed	
☐	☒	The exact sample size (*n*) for each experimental group/condition, given as a discrete number and unit of measurement
☐	☒	A statement on whether measurements were taken from distinct samples or whether the same sample was measured repeatedly
☐	☒	The statistical test(s) used AND whether they are one- or two-sided<br>*Only common tests should be described solely by name; describe more complex techniques in the Methods section.*
☐	☒	A description of all covariates tested
☐	☒	A description of any assumptions or corrections, such as tests of normality and adjustment for multiple comparisons
☐	☒	A full description of the statistical parameters including central tendency (e.g. means) or other basic estimates (e.g. regression coefficient) AND variation (e.g. standard deviation) or associated estimates of uncertainty (e.g. confidence intervals)
☐	☒	For null hypothesis testing, the test statistic (e.g. *F*, *t*, *r*) with confidence intervals, effect sizes, degrees of freedom and *P* value noted<br>*Give P values as exact values whenever suitable.*
☒	☐	For Bayesian analysis, information on the choice of priors and Markov chain Monte Carlo settings
☒	☐	For hierarchical and complex designs, identification of the appropriate level for tests and full reporting of outcomes
☒	☐	Estimates of effect sizes (e.g. Cohen's *d*, Pearson's *r*), indicating how they were calculated

Our web collection on statistics for biologists contains articles on many of the points above.

Software and code

Policy information about availability of computer code

Data collection	RNA-Seq was performed using Hi-Seq 2500 (Illumina) using a 100 cycle, single end protocol providing approximately 90 million reads per sample. Base call files were converted to fastq format using Bcl2Fastq (Illumina). In this study, Young Women's Breast Cancer (YWBC) datasets were collected from 8 studies and downloaded from the Gene Expression Omnibus (GEO) with the following accession number: GSE199266, GSE2062467, GSE2165368, GSE653269, GSE299073, GSE492270, GSE739071 and GSE1961572. The GEOquery and biomaRt R packages were used to download the raw expression and meta data. For IHC, stained sections were scanned digitally using Aperio ImageScope AT2 (Leica Biosystems, CA, USA) at 20x magnification utilizing the Aperio ScanScope software. RNA expression data for healthy nulliparous and healthy postpartum tissues were downloaded from GEO with the following accession number GSE26457.
Data analysis	All RNA Seq reads were aligned to the human reference genome (GRCh38, release 84) using STAR (version 2.5.2b) with default parameters. The STAR "GeneCounts" module was used to quantify the number of reads mapping to each gene. We also used RSEM (version: v1.2.31) to quantify per kilobase of transcript per million (FPKM) of the gene expressions. Gene expressions quantified by read counts from STAR were used as input into DESeq2.VST transformed counts were obtained within DESeq2. Hierarchical clustering of the z-score transformed Euclidean average linkage distances through the Morpheus software package (https://software.broadinstitute.org/morpheus). For Gene Set Enrichment Analysis (GSEA) on PPBC compared to NPBC, GSEA version 4.0.3. was used identify enriched gene sets from the Molecular Signature Database (MSigDB v 7.0, Hallmark, Collection 2,3,5-7), as well as 100 customized gene sets prepared from studies relevant to breast cancer, cancer immunity and normal breast biology which are provided, referenced and annotated in supplemental data 01. In order to infer the activities of transcription factors, we used the master regulator inference algorithm (MARINa) compiled in R 'viper' package to perform the regulon analyses on PPBC and NPBC samples. We employed MiXCR (Version 3.0.12,MiLaboratory LLC) to analyze TCR. In order to estimate the abundances of immune cells from the bulk RNA-seq, we utilized CIBERSORT63 to calculate the proportions of 22 human leukocyte cell subsets defined in the CIBERSORT package for each bulk RNA-seq sampleFor the Young Womens' Breast Cancer datasets raw datasets with different Affymetrix platforms were merged together and the expressions of all datasets were corrected by ComBat R package to remove the underlying batch effects. For IHC, all semi-quantitative image analysis was performed using Aperio deconvolution algorithms (Aperio-ImageScope Software v 12.4.3., Leica Biosystems, CA, USA). Further, Image processing including selection of region of interest, alignment of selected regions and extraction of AEC signals was performed in MATLAB version V9.90.1592791 using the SURF algorithm in the Computer

Vision Toolbox (The MathWorks, Inc) and FIJI (v 2.1). Cell quantification for multiplex IHC also included use of CellProfiler Version 4.1.3 and FCS Express Image Cytometry RUO (7.06.0015, De Novo Software, Glendale, CA). Statistical significance determine by p-values were generated by GraphPad-Prism Software (V9.2.0) (GraphPad Software, San Diego, California USA). Whole exome gene expression array data from healthy nulliparous (NP, blue, n=30) or postpartum breast (PP, red, within 2 years of completed pregnancy, n= 10) tissues was obtained from a previous study (GEO-GSE26457). Data was normalized utilizing Transcriptome Analysis Console software (V.4.02, ThermoFisher Scientific)

For manuscripts utilizing custom algorithms or software that are central to the research but not yet described in published literature, software must be made available to editors and reviewers. We strongly encourage code deposition in a community repository (e.g. GitHub). See the Nature Research guidelines for submitting code & software for further information.

Data

Policy information about availability of data

All manuscripts must include a data availability statement. This statement should provide the following information, where applicable:

- Accession codes, unique identifiers, or web links for publicly available datasets
- A list of figures that have associated raw data
- A description of any restrictions on data availability

RNA derived sequencing data generated in this study has been deposited in the Gene Expression Omnibus (GEO) database under accession code GSE158854 [https://www.ncbi.nlm.nih.gov/geo/query/acc.cgi?acc=GSE158854]. All numerical data used in generating plots of figures is available in Source Data. The publicly available RNA expression data from healthy nulliparous and postpartum breast tissues used in this study are available in the GEO database under accession code GSE26457 [https://www.ncbi.nlm.nih.gov/geo/query/acc.cgi?acc=GSE26457]. The publicly available outcomes data based upon RNA expression profiling used in this study as a young women's breast cancer cohort are available from the GEO database under accession codes, GSE1992 [https://www.ncbi.nlm.nih.gov/geo/query/acc.cgi?acc=GSE1992] , GSE20624 [https://www.ncbi.nlm.nih.gov/geo/query/acc.cgi?acc=GSE20624], GSE21653 [https://www.ncbi.nlm.nih.gov/geo/query/acc.cgi?acc=GSE21653], GSE6532 [https://www.ncbi.nlm.nih.gov/geo/query/acc.cgi?acc=GSE6532], GSE2990[https://www.ncbi.nlm.nih.gov/geo/query/acc.cgi?acc=GSE2990], GSE4922 [https://www.ncbi.nlm.nih.gov/geo/query/acc.cgi?acc=GSE4922], GSE7390 [https://www.ncbi.nlm.nih.gov/geo/query/acc.cgi?acc=GSE7390] and GSE19615 [https://www.ncbi.nlm.nih.gov/geo/query/acc.cgi?acc=GSE19615]. All remaining data are available within the Article, Supplementary Information or Source Data files. All other data including IHC data generated by the authors and used for the composition of figures (IHC data figures 3-4, supplementary figure 2-3) is provided in its entirety in Source Data.

Field-specific reporting

Please select the one below that is the best fit for your research. If you are not sure, read the appropriate sections before making your selection.

☒ Life sciences ☐ Behavioural & social sciences ☐ Ecological, evolutionary & environmental sciences

For a reference copy of the document with all sections, see nature.com/documents/nr-reporting-summary-flat.pdf

Life sciences study design

All studies must disclose on these points even when the disclosure is negative.

Sample size	In this study, 7 nulliparous and 9 postpartum breast cancer FFPE specimens were examined for the determination of differences in RNA expression profiles by RNA Seq. These represented all clinically annotated ER+ cases of sufficient RNA quality from 40 extracted specimens all of which were pre-selected to have matching parity data. Samples size was dictated solely based upon sample availability, sample characteristics (ER status) and necessary quality of extracted RNA. All available samples meeting these criteria for both cohorts were sequenced. Based upon previous published studies comparing ER+ to ER- FFPE specimens in which n=3 was sufficient for unbiased cluster separation of samples by ER status based upon RNA expression profiling (https://doi.org/10.1186/s12920-019-0643-z) the available n=7 for NPBC and n=9 for PPBC were deemed sufficiently powered for an RNA expression profiling study.
Data exclusions	Prior to sequencing, chart review and sample selection were conducted to generate a balance cohort, matched for age, stage of disease, clinically determine ER status and RNA extraction quality. No data exclusions were predetermined or applied for RNA-Seq . In IHC validation, not data was excluded and represents the totality of all completed datasets. However not all samples passed QC standards for all analytes required for alignment and execution of the multiplex pipeline, consequently multiplex cohorts differed in number (PPBC n =14, NPBC n=13) from single analyte IHC results (PPBC n=15, NPBC n=15).
Replication	To test reproducibility and relevance of expression signature found from the comparison between the 7 nulliparous and 9 postpartum breast cancer case expression profiles we compiled a Young Women's Breast Cancer (YWBC) dataset collected from 8 studies and downloaded from the Gene Expression Omnibus (GEO) with the following accession number: GSE199266, GSE2062467, GSE2165368, GSE653269, GSE299073, GSE492270, GSE739071 and GSE1961572, and demonstrated that postpartum breast cancer signature likewise associated with poor outcome. Validation of expression profiling approaches was rigorously tested and previously reported (https://doi.org/10.1186/s12920-019-0643-z). All RNA isolation and sequencing was performed once per sample, with pooled libraries composed of both NPBC and PPBC specimens. Results were interrogated for evidence of lane bias with no statistical support found for such confounders. All IHC data acquisition was performed on multiple ROI per sample to avoid ROI selection bias, but segregated based upon ROI location (tumor border vs intratumoral) Data presented is the average of all ROI's acquired for that sample based upon locality. The pipeline for multiplex IHC data acquisition and analysis was been previously validated and reported.
Randomization	Samples were allocated into groups based upon parity status for determination of common characteristic of postpartum breast cancer, the chief objective of this study. However multiple clustering analyses were performed that were agnostic to parity status and resulting groupings are displayed in the manuscript. For sequencing, samples were randomized and reviewed to insure composition of both nulliparous and postpartum samples to control for lane, library prep and chip bias's in sequencing.

Blinding	FFPE specimens were submitted for extraction and RNA sequencing completely blinded for purposes of raw data acquisition, mapping and normalization. Likewise IHC acquisition and single cell analyses was carried out blinded for parity status and by two independent analysts. Sample identities and parity status were included as a non-dependent variables for cluster analysis (Figure 1), which was carried out with randomized sample number designations and operator independent matrix inputs. Samples and associated data were unblinded to conduct analyses intended to identify characteristics of a postpartum breast cancer (GSEA, Regulon, Cibersort) and to designate a PPBC signature and for composition of figures comparing features between PPBC and NPBC specimens, which requires the parity status of the samples to be known.

Reporting for specific materials, systems and methods

We require information from authors about some types of materials, experimental systems and methods used in many studies. Here, indicate whether each material, system or method listed is relevant to your study. If you are not sure if a list item applies to your research, read the appropriate section before selecting a response.

Materials & experimental systems

n/a	Involved in the study
☐	☒ Antibodies
☒	☐ Eukaryotic cell lines
☒	☐ Palaeontology and archaeology
☒	☐ Animals and other organisms
☐	☒ Human research participants
☒	☐ Clinical data
☒	☐ Dual use research of concern

Methods

n/a	Involved in the study
☒	☐ ChIP-seq
☒	☐ Flow cytometry
☒	☐ MRI-based neuroimaging

Antibodies

Antibodies used	IHC was conducted utilizing established and previously reported multiplex (serial) IHC methodologies with the following order, dilution and sources of antibodies. Cycle 1 (PD-1, abcam, ab52587, Clone NAT105, 1:100, 1Hr), Cycle 2 (Ki67, Thermofisher, RM-9106-S, Clone SP6, 1:300, 1Hr), Cycle 3 (TOX1, abcam, ab237009, Clone NAN448B, 1:800, overnight), Cycle 4 (p53, Thermofisher, MA5-12557, Clone DO-7, 1:100, 2Hr), Cycle 5 (Phospho-Histone H2A.X (Ser139), Cell Signaling, 9718, Clone 20E3, 1:250, 1Hr), Cycle 6 (CD8, BioSB, BSB5174, Clone C8/144B, 1:100, 1Hr), Cycle 7 (CD3, Dako, A0452, 1:400, overnight), Cycle 8 (CD45, Dako, M0701, Clones 2B11 + PD7/26, 1:300, 1Hr). Secondary anti-rabbit or anti-mouse Simple Stain MAX PO Histofine Peroxidase Polymer (Nichirei Biochemicals, 414144 or 414134) or anti-rat ImmPRESS Peroxidase Polymer (Vector Laboratories, MP-7444) antibodies were utilized according to manufacturer's protocols. ER staining was performed at 1:200 dilution for 1 Hr at room temperature. (Novocastra, NCL-L-ER-6F11).
Validation	All antibodies were validated and titrated on FFPE preserved tissue microarray or control tissues composed of the following human tissues, invasive breast cancer, normal breast involution tissue, tonsil, spleen, placenta, colon, prostate and liver. A tissue microarray was included on every stained slide to identify variations in staining and validate successful execution of IHC.

Human research participants

Policy information about studies involving human research participants

Population characteristics	As outlined in provided supplemental table 1a, collected sample covariate demographics included Age at Dx, Race, Ethnicity, BMI, Stage at Dx, Clinical IHC determination of; ER, PR and Her2 status, parity status and years post last child birth. Covariate considerations between postpartum and nulliparous breast cancer cohorts were evaluated (Supplemental Table 1b) with no statistical differences except age at Dx (p=0.04) with nulliparous individuals averaging 5 years older at 39.1 years old at time of diagnosis.
Recruitment	The tissues evaluated in this study are comprised of clinical sample from clinical archives obtained from women less than or equal to 45 years of age when diagnosed with invasive cancer who were receiving standard of care treatment. All FFPE samples came from treatment naive specimens at time of initial diagnosis. All clinical data and specimens provided to the laboratory research team were labeled only with study-specific identifiers at all points, and this study was given a waiver of informed consent by the participating IRBs. As detailed in the manuscript, samples were selected based upon chart reviewed characteristics providing certainty of a primary breast cancer diagnosis, breast cancer attributes, age and parity status. All samples from all races and ethnicities and body sizes meeting these samples specific criteria were included and characteristics recorded in the provided table. Breast cancer specimens from biological males were excluded from the nulliparous cohort to reduce confounding variables of biological signals. For the purposes of this study only ER+ specimens with detailed parity data were considered. These criteria were decided upon in order to reduce variables outside of parity status that could increase variation in RNA expression profiles, and impair the ability to obtain the chief objective of this study which is dependent upon a relatively small cohort. The results of the RNA seq are bolstered by the increased availability of adequate samples for IHC, which were consistent with those obtained by RNA Seq and the associated specimens. Due to the highly selected nature of these samples it is uncertain how long the identified postpartum influence persists, nor how the identified characteristics in ER+ PPBC compare to ER+ NPBC individuals compares to postmenopausal and other cancer subtypes (i.e. ER-)

Ethics oversight	The research was conducted on archived FFPE tissues samples collected under IRB approved protocols at the Kaiser Permanente Northwest Center for Health Research and the Oregon Health & Science University.

Note that full information on the approval of the study protocol must also be provided in the manuscript.
